# High-density electromyographic data during isometric contractions of the ankle joint in children with cerebral palsy pre and post BoNT-A treatment

**DOI:** 10.1016/j.dib.2019.103840

**Published:** 2019-03-21

**Authors:** Lukas Gerald Wiedemann, Sarah Ward, Eewei Lim, Nichola Carolyn Wilson, Amy Hogan, Aleš Holobar, Andrew McDaid

**Affiliations:** a Department of Mechanical Engineering, The University of Auckland, Auckland, 1010, New Zealand; b University College Dublin, School of Public Health, Physiotherapy & Sport Science, Dublin 4, Ireland; c Starship Children's Health, Auckland, 1010, New Zealand; d Cerebral Palsy Society of New Zealand, Auckland, 1010, New Zealand; e Faculty of Electrical Engineering and Computer Science, University of Maribor, Maribor, 2000, Slovenia

## Abstract

Understanding the underlying mechanisms leading to progressive muscle pathologies in spastic Cerebral Palsy remains a challenging field of research. Furthermore, Botulinum Neurotoxin-A (BoNT-A) is a frequent intervention to treat spasticity in CP but its effects on neuromuscular properties are not yet fully explored. High-density Electromyographic (HD-EMG) data have been collected before and after BoNT-A injections from children aged 5–15 years during isometric contractions of the ankle joint together with torque output, clinical assessments and demographic details. Data collected from a total of 13 children with and 29 children without spastic CP allow for between-group comparisons and are made available using Mendeley Data (https://doi.org/10.17632/3sbptrk54c.2 and https://doi.org/10.17632/3b98g5fyff.1).

**Table:** Specifications table

Subject area	Biomedical Sciences
More specific subject area	Biomechanics in Cerebral Palsy
Type of data	Excel file (.xlsx) and Matlab files (.mat)
How data was acquired	High-density Electromyography (BioSemi B·V., ActiveTwo Amsterdam, Netherlands), torque measurements (FUTEK, TFF350 1300in-lb, CA, USA), clinical assessment (Modified Ashworth Scale, Modified Tardieu Scale, Timed-up and go)
Data format	Raw, synchronised and interpolated
Experimental factors	Participants were familiarised with the experimental protocol by testing the equipment and software prior to recording
Experimental features	Participants performed isometric contractions of the ankle joint with real-time force feedback provided on a computer screen. High-density Electromyography and torque data were concurrently recorded during the voluntary contractions.
Data source location	Auckland, New Zealand
Data accessibility	The data is made available using Mendeley Data. Case group data: https://doi.org/10.17632/3sbptrk54c.2 Control group data: https://doi.org/10.17632/3b98g5fyff.1
Related research article	L. G. Wiedemann, V. R. Jayaneththi, J. Kimpton, A. Chan, M. A. Müller, A. Hogan, E. Lim, N. C. Wilson, and A. J. Mcdaid, “Neuromuscular characterisation in Cerebral Palsy using hybrid Hill-type models on isometric contractions,” *Comput. Biol. Med.*, vol. 103, no. September, pp. 269–276, 2018 [1].

**Table:** 

**Value of the data** • HD-EMG data can be used to evaluate muscle activation in Cerebral Palsy on a motor unit level. • The longitudinal data available for children with Cerebral Palsy facilitates evaluating the effect of Botulinum Neurotoxin-A treatment on muscle activation on a motor unit level. • The torque data available allows for the investigation of force coordination and strength characteristics (e.g. rapid force development and impulse) in children with and without CP. • Data was collected from age- and gender-matched typically developed children. • The data available for both limbs in some participants of the TD group allows for between limb comparisons in TD children.

## 1 Data

The dataset provided with this article is the first instance of high-density electromyographic (HD-EMG) data collected during isometric contractions of the ankle joint from children with and without spastic CP, before and 2–6 weeks after BoNT-A treatment. In addition, the concurrent torque data provides valuable information about the muscle force output. This will enable comparisons of muscle activation on a motor unit level pre- and post BoNT-A injections.

Furthermore, data on participants performing the Timed Up and Go test (TUG) are provided. This popular test can be attributed to the International Classification of Functioning, Disability and Health (ICF) domain ‘activity’ and provides important information about the participants' dynamic balance [2]. Subsequently, the TUG assessment can be used to evaluate relationships between neuromuscular characteristics and mobility function in CP. Additionally, the data collected from typically developed children enables between-group comparisons.

## 2 Experimental design, materials, and methods

### 2.1 Participants

Data was collected from children aged 5–15 years with spastic CP (CP group; n = 13) and typically developed children (TD group; n = 29). Inclusion criteria for the CP group included spasticity affecting the lower limb requiring BoNT-A treatment. Exclusion criteria for the CP group included severe cognitive or visual impairments interfering with the ability to cooperate with the experimental protocol, concomitant neurological diseases, BoNT-A injection within the previous 4 months, neurosurgical and lower-limb orthopaedics procedures within the previous 12-months, previous selective dorsal rhizotomy and/or intrathecal baclofen treatment. Children in the TD group were required to be healthy without neurosurgical or lower-limb orthopaedics procedures within the 12-months prior to participation. All participants and their parents or legal guardian provided written informed assent/consent and the measurements were conducted following the guidelines outlined by the Southern Health and Disability Ethics Committee, New Zealand (HDEC; Ethics reference: 17/STH/215).

In the CP group the limb tested was either the more affected leg with spasticity as assessed with the Modified Ashworth Scale, or the more dominant leg in cases were both limbs were similarly affected. Assessing the dominant side in case of equal spasticity in both legs was chosen because it was assumed that children will be more likely to complete the experimental protocol.

In two participants with CP both limbs were assessed. Individuals recruited for the TD group were gender- and age-matched within ±12-months to children in the CP group. For the TD group either both limbs were tested, or the limb assessed would depend on the limb tested for the gender- and age-matched individual in the CP group (i.e. either dominant or non-dominant leg). As a result, the TD group was recruited after the CP group. Children with CP performed the same measurements before (n = 13) and 2–6 weeks after (n = 12) they received BoNT-A treatment in their lower limb(s) affected with spasticity. In contrast, the TD group was tested only once. Therefore, the dataset allows for age-and gender-matched comparisons, between-limb difference analyses in the TD group, or pre-post comparisons in the CP group.

Demographic details (age, gender, height, weight, BMI and body side assessed) of all participants can be obtained from the data available with Mendeley Data [3], [4].

### 2.2 Clinical data

The clinical data collected from the children with CP are the Gross Motor Function Classification Score (GMFCS), the CP involvement (i.e. unilateral or bilateral), Modified Tardieu Scale (MTS), Modified Ashworth Scale (MAS), BoNT-A doses in Gastrocnemius, the most recent BoNT-A injection date after the pre-assessment, the previous BoNT-A injection date before the pre-assessment and the total number of BoNT-A treatments during lifespan prior to the data collection. Further details regarding the BoNT-A treatment are provided in Section 2.4.

### 2.3 Experimental protocol

**Fig. 1:**
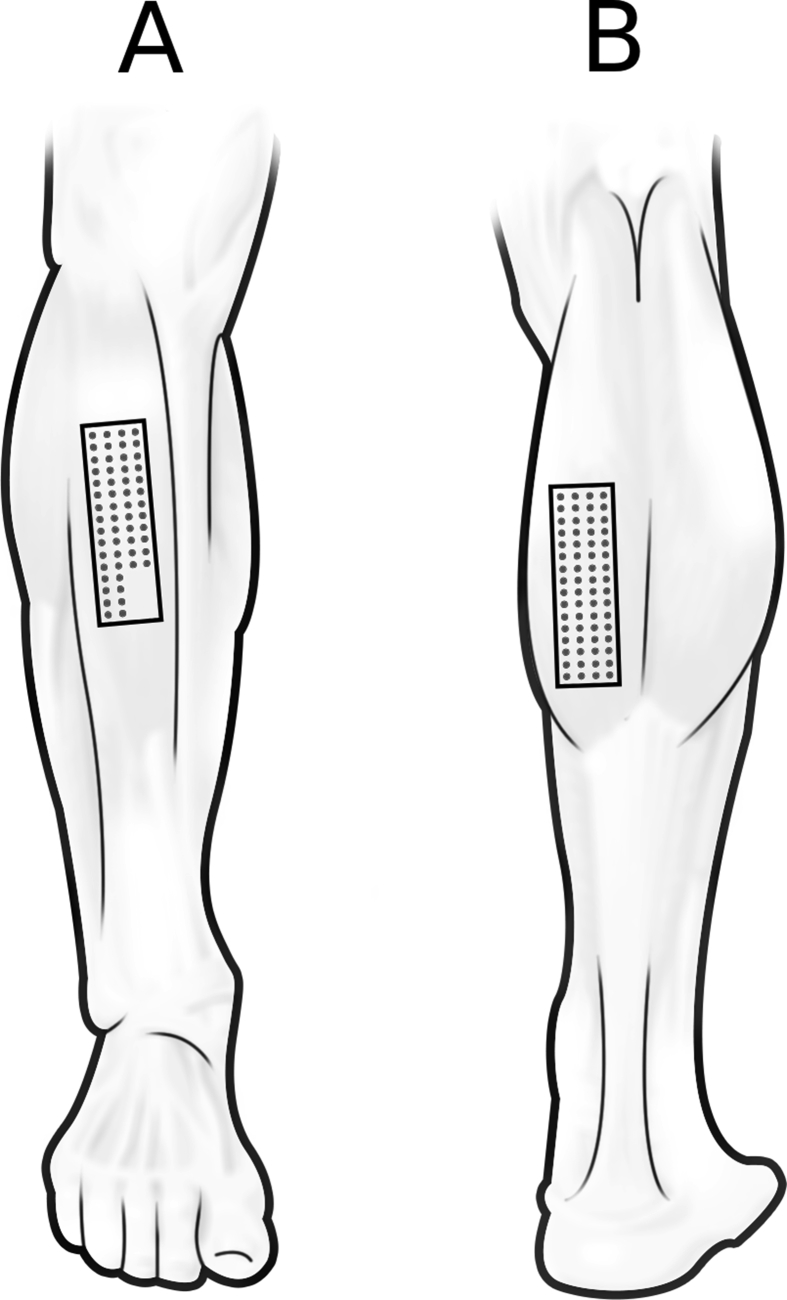
Electrode placement on the Tibialis anterior (A) and Gastrocnemius medialis (B).

Participants performed a Timed Up and Go test (TUG). The TUG begins seated in a chair, participants then stand up, walk 3-m, turn 180° around a marker (e.g. a cone), then walk back to the chair and sit down [5]. Participants were allowed one familiarization trial before they were timed to complete the test. Following the TUG test, HD-EMG signals were recorded using a BioSemi ActiveTwo system (BioSemi B·V., Amsterdam, Netherlands) with a sample frequency of 2048 Hz. FPC electrode arrays with 64 electrodes each (4 × 16 channels; FlexiMap, Auckland, New Zealand [6]) were applied on the skin above the medial head of the Gastrocnemius and the Tibialis anterior (Fig. 1) after a skin preparation gel was applied (Nuprep, Colorado, USA). The long side of the EMG array was oriented parallel with the muscle fibre direction. The centre of the electrode array was located according to the recommendations of SENIAM [7]. Interelectrode distance was 4 mm, the diameter of the gold-plated electrodes equalled 0.5 mm. The dry electrode array was applied using laser-cut double-sided foam tape (1.6 mm thickness, 3 M, USA) and conductive paste (Ten 20 Conductive Paste, Colorado, USA) [8]. The last four channels of the third and fourth column of the HD-EMG grid on the Tibialis anterior were not amplified due to a maximum of 120 amplified channels of the BioSemi system resulting in 56 channels recorded for this muscle.

**Fig. 2:**
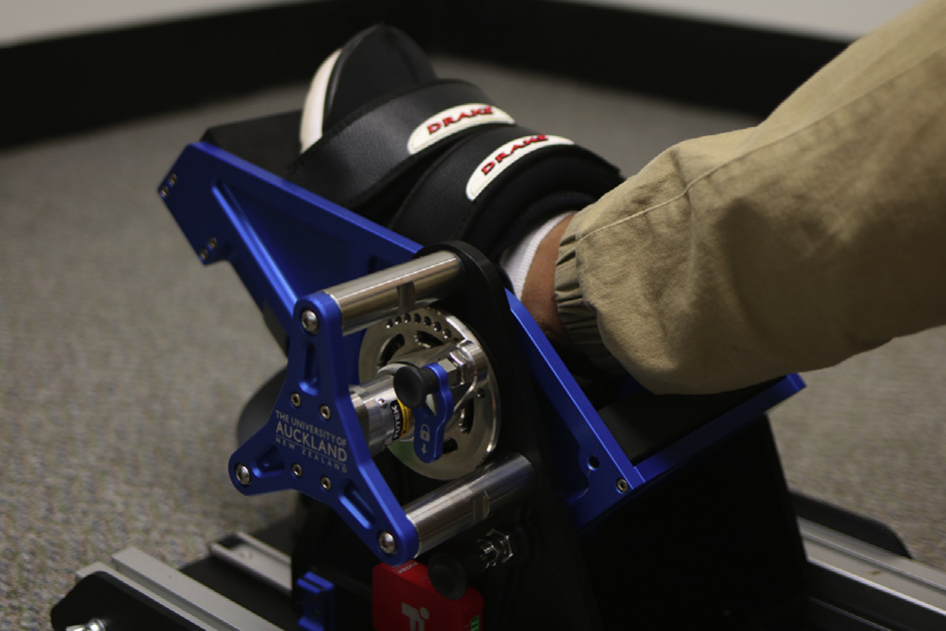
Footplate measuring the torque around the ankle joint during isometric contractions.

**Fig. 3:**
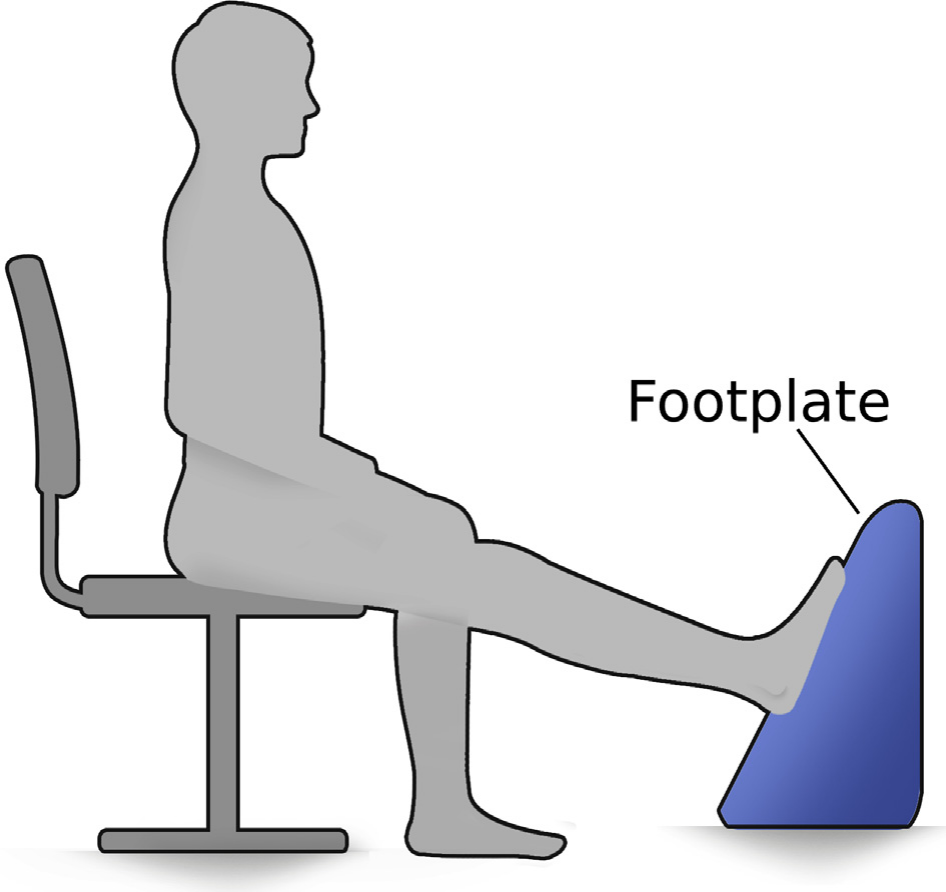
Participant posture during the isometric contractions.

**Fig. 4:**
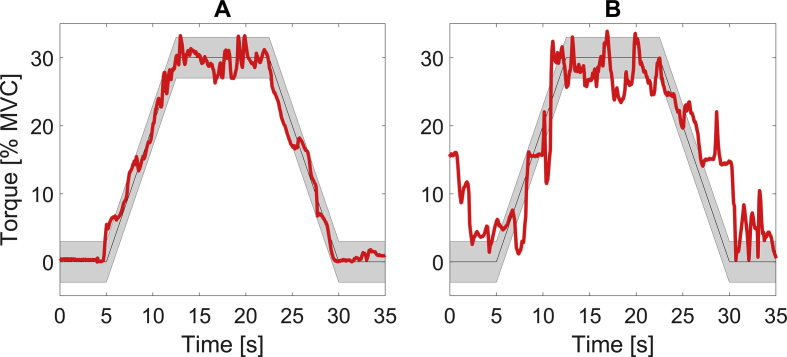
Two examples of torque trajectories in red colour of a child without (A) and with spastic CP (B) during a medium force ramp. The vertical distance between the two blue feedback lines equals 20% of the target torque at the plateau region.

For the voluntary force recordings participants sat upright on a chair. The lower limb was strapped into the testing device (Fig. 2), which recorded the torque around the ankle joint with a sample frequency of 500 Hz simultaneously with the HD-EMG system. As can be seen in Fig. 3 participants were instructed to keep the knee of the leg attached to the footplate extended throughout the recordings and the extension of the knee was visually inspected. The hip position varied from subject to subject in order to achieve as close a full knee extension as possible. As a limitation, the knee and hip angle were not recorded during the trials. The angle of the device was adjusted so that the participants’ ankle joint angle was 0° (i.e. neutral position) as measured with a plastic goniometer. After the participant was secured into the device two maximum voluntary isometric contractions (MVIC) of the ankle dorsiflexors and two for the plantarflexors were performed, each with a duration of >3 s with 3 min breaks between trials to avoid fatigue [9]. Following the MVICs, the participant performed isometric force ramps for the plantarflexors while receiving visual real-time torque feedback on a computer screen (Figs. 4, Video 1). Custom-made software was used to display the force feedback lines (Labview, National Instruments, Austin, TX, USA). Participants were familiarised with the force ramp trials until they felt confident with the experimental protocol prior to recording. A total of three low, three medium and two high isometric force ramps (in this sequence) were recorded for plantarflexion. These isometric force ramps consisted of a ramp-up phase (7.5 s), a plateau region (10 s) and a ramp-down phase (7.5 s) and differed in the contraction level of the plateau phase (Table 1). No force ramps for ankle dorsiflexion were recorded to limit the duration of the entire experimental protocol to approximately 1 h.

**Table 1:** Isometric force ramp characteristics.

	Contraction level at the plateau region	Breaks between trials	Absolute change in force during ramp phases
Low force ramps	15% MVIC	1 minute	2% MVIC s^−1^
Medium force ramps	30% MVIC	2 minutes	4% MVIC s^−1^
High force ramps	70% MVIC	3 minutes	9.33% MVIC s^−1^

Supplementary data related to this article can be found online at https://doi.org/10.1016/j.dib.2019.103840

The following are the Supplementary data related to this article:

Video 1 Demonstration of the real-time torque feedback on the computer screen. The blue lines illustrate the force ramp protocol and the participants were asked to stay within as accurately as possible. The red line represents the participant’s torque. In order to keep the children motivated during the trials and to avoid boredom the participants were able to choose between three different modes: a racing car, a space ship and a monkey.2 Video 1

The duration of 10s of the plateau phase is needed to ensure a sufficient time support for decomposing the HD-EMG signals into their motor unit firing patterns [10], [11]. The phase up and down length of 7.5s equated to a change in force of 2% MVIC s^−1^ for the low contraction ramp, which was in accordance with [9] to facilitate MU threshold analysis (i.e. the contraction level MUs are activated and de-activated). The change in force of 4% MVIC s^−1^ and 9.33% MVIC s^−1^ for the medium and high isometric force ramps, respectively, have not been reported in the literature and present novel data for analyzing the performance of decomposition methods regarding MU threshold analysis.

Most participants were able to comply with all measurements. The isometric force ramps could be completed by all participants except ‘noCP2_ankle5’ who performed two low and medium force ramps and one high isometric force ramp due to a lack of motivation. Just one MVIC for the plantarflexors was completed by participants ‘CP6_ankle’, ‘noCP_ankle24′ and ‘noCP2_ankle5’. Only one MVIC for the dorsiflexors was completed by ‘CP_ankle3′, ‘CP_ankle3_post’, ‘CP_ankle9_post’ and ‘CP_ankle11_post’ all due to poor motivation. Participants ‘CP_ankle6′ and ‘CP_ankle9′ did not perform any MVICs for the dorsiflexors because they were not able to pull with their toes.

It is further important to note, that in participant ‘CP_ankle01’ the incorrect leg was assessed post BoNT-A. Also, there was no post measurement for subject ‘CP_ankle13’.

### 2.4 BoNT-A injection procedure

Children with spastic CP received BoNT-A treatment as part of the standard clinical care. The BoNT-A preparation Botox (Allergan) was used in all children with CP. It is supplied as a vacuum-dried, preservative-free white powder in a vial containing 100IU of BoNT-A. Each vial was mixed with 2ml of 0.9% sodium chloride (normal saline). BoNT-A doses and sites of injection were determined by the physiotherapist and rehabilitation paediatrician, who assessed each child and determined individual clinical needs and goals while accounting for their weight. A course of treatment involved as many individual injections as was clinically indicated by the clinicians. The procedure was undertaken under a general anaesthetic in all cases. The BoNT-A was injected with a 25-gauge needle under ultrasound guidance using a portable ultrasound machine operated by the paediatrician injector.

Only participants ‘CP_ankle01’, ‘CP_ankle06’ and ‘CP_ankle10’ had multilevel lower limb Botox. The remaining participants with CP had single level lower limb Botox. Furthermore, participant ‘CP_ankle01’ had include injections into the upper limb as well.

In the interim 2–6 weeks after treatment and before the second assessment, all participants with CP received standard physiotherapy and were required to wear AFOs. Participant ‘CP_ankle06’ had a period of serial casting over two weeks.

### 2.5 Data pre-processing

The torque data were interpolated using the ‘interp1’ function and ‘spline’ method in Matlab (version R2018a, The Mathworks Inc., Natick, MA, USA) to match the sample frequency of the HD-EMG signals. Monopolar HD-EMG signals have not been digitally filtered and are provided in raw format as a. mat file. The first 16 rows in the. mat file represent the first column of the HD-EMG grid; rows 17–32 represent the second column; rows 33–48 contain the signals of the third column and rows 49–64 represent the fourth column). The EMG data are provided in μV and the unit of the torque data is Nm.

## References

[1] Wiedemann L.G., Jayaneththi V.R., Kimpton J., Chan A., Müller M.A., Hogan A., Lim E., Wilson N.C., Mcdaid A.J. (2018). Neuromuscular characterisation in Cerebral Palsy using hybrid Hill-type models on isometric contractions. Comput. Biol. Med..

[2] Saether R., Helbostad J.L., Riphagen I.I., Vik T. (2013). Clinical tools to assess balance in children and adults with cerebral palsy: a systematic review. Dev. Med. Child Neurol..

[3] Wiedemann L., Ward S., Lim E., Wilson N., Hogan A., Holobar A., McDaid A. (2018).

[4] Wiedemann L., Ward S., Lim E., Wilson N., Hogan A., Holobar A., McDaid A. (2018).

[5] Podsiadlo D., Richardson S. (1991). “The timed ‘up & Go’: a test of basic functional mobility for frail elderly persons. J. Am. Geriatr. Soc..

[6] Peng D., O'Grady G., Egbuji J.U., Lammers W.J., Budjett D., Nielsen P., Windsor J.A., Pullan A.J., Cheng L.K. (2009). High-resolution mapping of in vivo gastrointestinal slow wave activity using flexible printed circuit board electrodes: methodology and validation. Ann. Biomed. Eng..

[7] Hermens H.J., Freriks B., Disselhorst-Klug C., Rau G. (2000). Development of recommendations for SEMG sensors and sensor placement procedures. J. Electromyogr. Kinesiol..

[8] Wiedemann L.G., Mcdaid A.J. (2017). On the function and robustness of skin-electrode interfaces for high-density electromyography: towards ubiquitous integration with robotics devices. In 2017 IEEE Life Sciences Conference (LSC).

[9] Watanabe K., Gazzoni M., Holobar A., Miyamoto T., Fukuda K., Merletti R., Moritani T. (Nov. 2013). Motor unit firing pattern of vastus lateralis muscle in type 2 diabetes mellitus patients. Muscle Nerve.

[10] Holobar A., Zazula D. (2007). Multichannel blind source separation using convolution kernel compensation. IEEE Trans. Signal Process..

[11] Holobar A., Farina D. (2014). Blind source identification from the multichannel surface electromyogram. Physiol. Meas..

